# Conference at Birmingham

**Published:** 1912-09-28

**Authors:** 


### THE BRITISH HOSPITALS ASSOCIATION.

### CONFERENCE AT BIRMINGHAM.

### Full Report of the Proceedings and Discussions.

The third annual Conference of the British Hospitals Association was held at Birmingham on September 19 and 20. By the permission of the Lord Mayor (Alderman W. H. Bowater) the meetings were held in the Council Chamber. The Lord Mayor attended the opening proceedings and gave the visitors a very cordial welcome to the city. The following is a list of those present:—

Mr. Hugh C. Aston, Women's Hospital, Birmingham; Dr. Auden, Education Department, Birmingham.

Mr. T. Foley Bache, District Hospital, West Bromwich; Mr. John W. Barnes, Royal Infirmary, Sheffield; Mr. J. J. Barron, Royal Infirmary, Bradford; Mr. Charles Beech, District Hospital, West Bromwich; Mr. E. Bettridge, General Dispensary, Birmingham; Mr. Thomas E. Bladon, General Dispensary, Birmingham; Rev. E. Braimbridge, Infirmary and Children's Hospital, Kidderminster; Mr. Fred J. Bray, General Infirmary, Leeds; Mr. W. Bray, Brownlow Hill Workhouse, Liverpool; Mr. E. Montague Browne, General Hospital, Northampton; Councillor W. Brown, Skin Hospital, Birmingham; Mr. James Bruce, Leith Hospital; Mr. A. W. Bryant, Skin Hospital, Birmingham; Mr. J. Courtney Buchanan, Metropolitan Hospital, London; Mr. R. F. L. Burton, Salop Infirmary, Shrewsbury.

Rev. G. Montgomery Campbell, Dumfries and Galloway Royal Infirmary; Mr. Albert Cay, Warneford Hospital, Leamington; Mr. J. B. Clarke, General Hospital, Birmingham (President); Mr. R. D. Clarkson, M.D., Scottish National Institution for Education of Imbecile Children; Mr. Howard J. Collins, General Hospital, Birmingham (local Conference Secretary); Mr. J. H. Cooke, Albert Infirmary, Winsford; Rev. G. B. Cronshaw, Radcliffe Infirmary, Oxford; Mr. W. A. Crosbee, Queen's Hospital, Birmingham.

Mr. J. A. Deeley, Women's Hospital, Wolverhampton; Mr. Wm. Dewar, Hospital of St. Cross, Rugby; Mr. John Dunn, Victoria Infirmary, Glasgow.

Canon Evans, Coventry and Warwickshire Hospital.

Lieut.-Col. Sir Joseph Fayer, Royal Infirmary, Edinburgh; Mr. E. Forrest, General Dispensary, Birmingham; Mr. E. Forster, Royal Infirmary, Derby; Mr. Gerald Friend, St. George's Hospital, London; Mr. George J. Furness, Passmore Edwards' Cottage Hospital for Willesden; Mr. F. W. Francis, Warneford Hospital, Leamington.

Captain E. T. Garston, St. George's Hospital, London; Mr. Herbert Gill, Royal Infirmary, Bradford; Mr. Arthur Griffiths, East Suffolk and Ipswich Hospital; Mr. W. Grisewood, St. George's Hospital, London; Mr. S. G. Grew, Ear and Throat Hospital, Birmingham; Mr. S. D. Gloss, London.

Mr. Frank I. Hancock, District Hospital, West Bromwich; Mr. W. H. Harper, General Hospital, Wolverhampton; Mr. A. Hastings, Children's Hospital, Birmingham; Mr. W. H. Head, General Hospital, Cheltenham; Mr. Howard Heaton, Orthopædic Hospital, Birmingham; Mr. E. Shaw Helier, General Hospital, Wolverhampton; Mr. Edward Hemingway, General Infirmary, Dewsbury; Mr. G. H. Hume, Royal Victoria Infirmary.

Mr. Edward Iliffe, Coventry and Warwickshire Hospital.

Dr. James, London; Mr. Stewart Johnson, Hospital for Sick Children, London (Hon. Treasurer); Mr. A. J. Johnston, Coventry and Warwickshire Hospital.

Mr. William Kay, Infirmary, Bolton; Mr. Edgar S. Kemp, Hospital Almoners' Council, London; Mr. Richard Kershaw, Throat and General Hospital, London, W.C.

Mr. Eustace Lees, Eye Infirmary, Wolverhampton; Mr. Conway Lowe, Eye Hospital, Birmingham.

Dr. D. O. Macgregor, Victoria Infirmary, Glasgow; Dr. D. J. Mackintosh, Western Infirmary, Glasgow (Hon. Secretary); Mr. Thomas Maddock, Cottage Hospital, Wellingborough; Mr. A. Mansell, Salop Infirmary, Shrewsbury; Mr. C. A. Mason, Eye Hospital, Birmingham; Mr. Rowland Mason, Skin Hospital, Birmingham; Mr. H. R. Maynard, King Edward's Hospital Fund, London; Mr. F. Messent, East Suffolk and Ipswich Hospital.

Mr. Allen Naldrett, Royal Southern Hospital, Liverpool; Mr. Fredk. Neden, County Hospital, York; Mr. John Nettlefold, Women's Hospital, Birmingham; Mr. J. Stephen Neil, General Hospital, Wolverhampton; Mr. J. Nicholls, Guest Hospital, Dudley; Mr. G. A. Nutt, City Aid Society, Birmingham.

Mr. Frank Olwer, Infirmary, Ashton-under-Lyne; Mr. R. A. Owthwaite, General Hospital, Hampstead, N.W.

Mr. W. Pearson, Gravesend Hospital; Mr. W. Precey, District Hospital, Walsall; Mr. Samuel Price, Children's Hospital, Birmingham; Mr. J. Danvers Power, King Edward's Hospital Fund, London.

Mr. Sidney M. Quennell, Westminster Hospital, London, S.W.

Dr. Nathan Raw, Mill Road Infirmary, Liverpool; Mr. T. Basil Rhodes, North Staffordshire Infirmary, Stoke-on-Trent; Mr. Carrington S. Risbee, General Hospital, Northampton; Mr. John H. Robinson, Miller General Hospital, S.E.; Colonel J. A. Roxburgh, Western Infirmary, Glasgow; Mr. J. A. Rudd, Coventry and Warwickshire Hospital.

Mr. J. H. Shaw, Infirmary, Southport; Mr. Thomas C. Shingler, Wenlock and Broseley Hospitals; Mr. James Henry Smethurst, Infirmary, Warrington; Mr. Fred Smith, Warneford Hospital, Leamington; Dr. E. Gilbert Smith, Skin Hospital, Birmingham; Mr. G. Shrewsbury Smith, General Infirmary, Worcester; Mr. J. E. Smith, Hospital for Women and Children, Bristol; Mr. H. D. Stephenson, Gravesend Hospital; Mr. W. Stevenson, Devonshire Hospital, Buxton; Rev. H. V. Stuart, North Staffordshire Infirmary; Mr. H. Sumner, Skin Hospital, Birmingham; Mr. W. Spruce, G.W.R. Medical Fund Society.

Mr. Conrad W. Thies, Royal Free Hospital, London (Hon. Treasurer); Lady Thomas, Newport and Monmouthshire Hospital; Sir Garrod Thomas, M.D., Newport and Monmouthshire Hospital; Mr. Ben B. Tilley, General Hospital, Birmingham; Mr. R. C. Trigger, North Staffordshire Infirmary; Mr. E. J. Thomas, Ear and Throat Hospital, Birmingham.

Mr. A. William West (Chairman of Council); Mr. Harold Wigg, District Hospital, Walsall; Mr. S. N. Williams, District Hospital, West Bromwich; Mr. F. H. Webb, General Dispensary, Birmingham.

### LORD MAYOR'S WELCOME.

The Lord Mayor said: Before you commence the principal business of the Conference I should just like to avail myself of this opportunity of welcoming your Association to our city. You certainly deserve a civic welcome. I hope you will accept it from me on behalf of our citizens generally, of our City Council, and of the different committees and managers of our many hospitals. We have a great many hospitals in this city. You are experts and I should not like to speak of them as to their great merits, but you will have an opportunity of visiting any of them you think fit and forming your own opinion concerning them. To me this Association seems part of our municipal administration. We spend a great deal of money locally through our Health Committee, and a good part of it, some £50,000 or £60,000 per annum, is spent in the maintenance of the different civic hospitals. We realise that a municipal authority can never be better concerned than in looking after the public health of its citizens. It is not so many years ago that the first city hospital was founded, and we only then began to look after fever cases and smallpox when unfortunate epidemics existed; but now, as you know, there are some ten or twelve diseases that must be compulsorily notified to the public authorities here. After they are notified we not only just register them, but our staff and our many medical officers of health look after the cases afterwards. Many of them we actually treat in institutions, but we go further, and those for whom we have no institutions we visit in their homes and instruct them how the better to look after themselves. You will see, therefore, that municipalities are more and more taking into their charge the public health of the city. To me this is all pointing to the time when the communities will take the entire charge of the public treatment of the sick. It may be desirable to hasten that. Many people think it will be better to retard it, but I think the tendency is to more and more take hold of the hospitals, and whilst it will ever be in the power of those who have means to have private treatment of their illness, yet as a rule I think it will very soon be brought about that the State will take over by national means, or by what I would prefer—municipal agencies will take charge of all the hospitals. As regards national insurance against sickness, that is a step in the same direction. What action hospitals will take, I suppose, will depend upon circumstances. You cannot as yet say until you know what particular policy the doctors will adopt. I wish the Government would realise that they have a very strong public opinion in the direction of doctors being well paid. They do not want strict investigations made as to what is the lowest sum that would be a living wage for a well-educated doctor. No money can be better spent than in improving the general health of the community, and it behoves us to get the very best medical and surgical attention that is to be obtained. As regards the aims of this Association, they are the very opposite of selfish ones. There is a feeling between the managers of the different hospitals which is very different to what it is in most professions, and certainly in all businesses. If any committee or manager visits any other institution and says, "We find you can carry on the administration of your institution at a much less cost than we can, and your statistics prove that your results are more satisfactory than our own. Will you tell me everything so that we may put our house in the same order?" you welcome them. You have these meetings so that you may profit by each other's experience. You are very different to businesses. I wonder what answer a manufacturer would get if he went to a rival manufacturer and said, "How is it you can sell better than I can? How is it your goods are better than mine? Will you allow me to walk over your place and you can show where I am at fault?" I welcome a Conference like this, and I am sure very great benefit will arise from your deliberations.

The President (Mr. J. B. Clarke), Chairman of the Board of Management of the Birmingham General Hospital, who occupied a seat on the dais beside the Lord Mayor, in the name of the Council expressed their thanks to his Lordship. He (the President) agreed with the Lord Mayor that they had no selfish motives. The Association was representative of men in all parts of the Kingdom. The Lord Mayor briefly replied, and mentioned that their President was Chairman of the General Hospital, which received about 78,000 patients every year. It was greatly due to his business ability that the hospital was so successful.

His Lordship then left the room and the chair was taken by the President. He claimed that in Birmingham they had done something for the relief of the sick and the poor and the afflicted and he hoped they would appreciate the hospital work in the city. He regretted that Sir William Collins was prevented from attending to read his paper as he was attending an International Conference at Geneva. His paper (in which we have inserted cross-headings) would be read by Mr. Thies.

